# Determination of nitrite in rifapentine and analysis of the formation causes of nitrosamine impurity

**DOI:** 10.3389/fchem.2026.1791665

**Published:** 2026-03-17

**Authors:** Yaodong Ping, Yongxiang Liu, Hairuo Wen, Nie Wen, Bin Li, Yuan Chen, Ye Tian

**Affiliations:** 1 Department of Pharmacy, Peking University Cancer Hospital (Inner Mongolia Campus) & Affiliated Cancer Hospital of Inner Mongolia Medical University, Hohhot, China; 2 Key laboratory of Carcinogenesis and Translational Research (Ministry of Education/Beijing), Department of Pharmacy, Peking University Cancer Hospital & Institute, Beijing, China; 3 Institute for Safety Evaluation, National Institutes for Food and Drug Control, Beijing Key Laboratory for Safety Evaluation of Drugs, Beijing, China; 4 Shenyang Pharmaceutical University, Shenyang, China

**Keywords:** CPNP, nitrite ions, nitrosamine impurities, nitrosation pathway, rifapentine capsules, small-molecule drugs

## Abstract

**Introduction:**

During drug manufacturing, residual nitrosamine salts in the final formulation are a key contributor to the formation of genotoxic nitrosamine impurities. However, trace-level concentrations and strong matrix interference complicate nitrosamine salt detection; therefore, selecting an appropriate analytical method is crucial.

**Methods:**

We developed a valve-switching ion chromatography method and performed method validation. We also conducted nitrification degradation experiments and validation studies of the nitrification pathway by detecting ^14^N-CPNP and ^15^N-CPNP using LC-HRMS after adding ^15^N-nitrite to rifaentine.

**Results and Discussion:**

The results of the method validation indicate its good linearity, accuracy, quantification, and detection limits. Nitrification degradation experiments demonstrate that CPNP in rifapentine can be generated via a nitrosation pathway. This pathway has also been validated by experimental results. This IC analytical method has the dual applications of genotoxic impurity quality control and elucidating impurity formation pathways in small-molecule drugs.

## 1 Introduction

According to the Global Tuberculosis Report in 2023, tuberculosis (TB) was the second leading cause of death from a single infectious agent in 2022, following COVID-19. In 2022, the number of confirmed TB cases worldwide reached 10.6 million, with 7.5 million new diagnoses and an estimated 1.3 million deaths annually. TB remains a global epidemic, with higher incidence rates in economically and culturally underdeveloped regions. Globally, China ranks third among the top 30 high-burden TB countries. Rifamycin antibiotics, particularly rifampin, are indispensable clinical treatments for TB, serving as first-line therapies with proven efficacy (WHO, 2023). However, in 2020, a World Health Organization study found genotoxic impurities, including 1-methyl-4-nitrosopiperazine and 1-cyclopentyl-4-nitrosopiperazine (CPNP), in both rifampin and rifapentine, which pose significant risks to patients with TB (WHO, 2024). Subsequently, the United States Food and Drug Administration set provisional limits for these impurities at 5 and 20 ppm, respectively.

**FIGURE 1:**
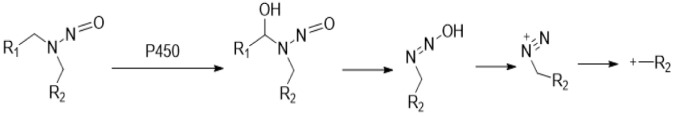
Metabolism of nitrosamines *in vivo*.

**FIGURE 2:**
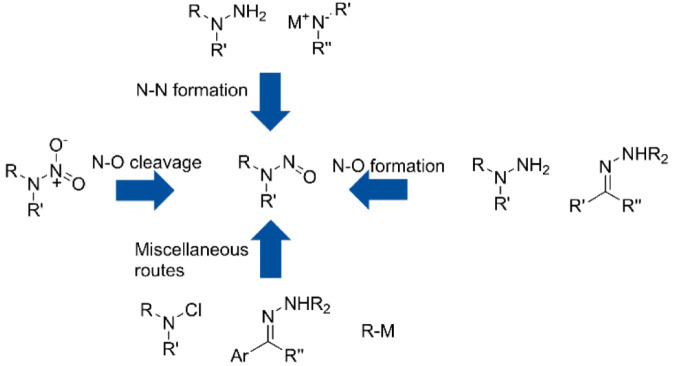
Formation pathway of nitrosamine impurities in chemicals.

Genotoxic impurities can directly or indirectly damage cellular DNA, causing mutations and carcinogenic risks at low concentrations (Figure 1) (Caishun et al., 2023). The detection of genotoxic nitrosamine impurities in pharmaceuticals has raised significant concerns. More than 120 nitrosamine compounds have been identified, with 75% carrying carcinogenic risks (Qian et al., 2021). Nitrosamine impurities form through various pathways, which can be broadly categorized into nitrosation and non-nitrosation reactions (Figure 2). Nitrosation involves the interaction of amine compounds with nitrosating agents, in which the hydrogen atoms of the compounds are replaced by nitroso groups. Nitrosating agents include nitrite ions, nitrosyl halides, nitrite esters, dinitrogen trioxide, and dinitrogen tetroxide. The use of amine compounds and sodium nitrite as nitrosating agents during the production of active pharmaceutical ingredients (APIs) can generate nitrosamine impurities (Qian et al., 2021). For example, in the production of metformin, dimethylamine reacts with sodium nitrite, which is used to quench excess sodium azide, leading to the formation of N-nitrosodimethylamine (Yuanchao et al., 2022). Similarly, N-nitrosodimethylamine is generated during sartan drug production following the reaction of amines with sodium nitrite (Nitrosamine Impurities in Human Medicinal, 2020). Consequently, monitoring or controlling nitrite levels in APIs or formulations is essential to prevent interactions between nitrosating agents and amine compounds and therefore reduce nitrosation reactions. The effective detection of nitrites in chemical pharmaceuticals is therefore a crucial research goal.

Common methods for detecting nitrite ions include spectrophotometry (Nini et al., 2023; Yanli et al., 2020), electrochemical methods (Turdean and Szabo, 2015), gas chromatography (Bei et al., 2022), liquid chromatography (Cheng et al., 2023; Lihua and Paibin, 2011), and ion chromatography (Heng et al., 2021; Xueting et al., 2020). Ion chromatography is widely used to detect ionic substances, including anions (Xiaohui et al., 2016). Compared with other methods, ion chromatography is more efficient, simple, and clean. The eluent generator requires only deionized water to produce the desired eluent concentration thereby enhancing detection sensitivity. Furthermore, when coupled with a suppressor, this approach markedly reduces background conductivity, improves analyte signals, and provides lower detection limits (Xiaosha et al., 2021). Although ion chromatography is ideal for detecting inorganic ions, the complex matrix of rifapentine capsules and the low polarity of the main component can lead to adsorption of the main component on the chromatographic column. Consequently, the sample cannot be directly injected into the ion chromatography column and requires pretreatment.

In this study, we established an improved ion chromatography method for detecting nitrite ions in rifapentine capsules. This method uses an NG1 column, which retains the main component but not inorganic ions and is coupled with column-switching technology to eliminate interference from the main component and matrix, thereby addressing co-elution issues in trace component detection (Yuexue et al., 2019). The proposed method offers high sensitivity, good linearity, high recovery rates, and low detection limits, and is suitable for detecting nitrite ions in drugs containing nitrosamine impurities.

## 2 Experimental

### 2.1 Materials and reagents

Magnesium stearate and pregelatinised starch are included as excipients in rifapentine capsules. Methanol and acetonitrile were purchased from Honeywell Burdick & Jackson (Charlotte, NC, United States). Nitrite standard solution was purchased from Guobiao (Beijing) Testing& Certification CO. (Beijing, China).

### 2.2 Instruments

The following instruments were used in this study: Thermo Dionex ICS-5000+ ion chromatograph. It consists of an eluent generator, conductivity detector, ASRS 300 suppressor, AS autosampler, and Chromelon software (ThermoFisher Scientific, Waltham, MA, United States). Thermo Dionex IonPac™ NG1,Thermo Dionex IonPac™ AG11-HC anion exchange column and Thermo Dionex IonPac™ AS11-HC anion exchange column were purchased from ThermoFisher Scientific (Waltham, MA, United States). Shimadzu LC 20AD liquid chromatograph fitted with a photo-diode array (PDA) detector and a LabSolution workstation (Shimadzu Corporation, Kyoto, Japan). An AB SCIEX 6500 Qtrap mass spectrometer (SCIEX Corporation, Framingham, MA, United States),a Shimadzu Shiseido Nanospace HPLC system with an Analyst workstation (Shimadzu Corporation, Kyoto, Japan), a Mettler Toledo XP205 electronic balance (Mettler-Toledo International Inc. , Zurich, Switzerland), and a ThermoScientific MAXQ 6000 shaker (ThermoFisher Scientific, Waltham, MA, United States). Thermo HPLC system fitted with a U3000 quaternary low-pressure gradient pump, autosampler, column oven and quadrupole detector (ThermoFisher Scientific, Waltham, MA, United States). A Q Exactive Focus High-Resolution Mass Spectrometer (ThermoFisher Scientific, Waltham, MA, United States).

### 2.3 Sample preparation

#### 2.3.1 Test solutions

The contents of a rifapentine capsule (equivalent to approximately 150 mg of rifapentine) were placed in a 50 mL centrifuge tube. Then, 7.8 mL methanol was added, and the tube was shaken for 0.5 min. Next, deionized water (7.2 mL) was added, and the tube was shaken for 10 min. Subsequently, the supernatant was filtered, and the filtrate was collected.

#### 2.3.2 Reference solutions

Appropriate amounts of the nitrite standard solution were diluted with 52% methanol to prepare reference solutions with concentrations of 5, 10, 25, 50, 100, and 200 ng/mL.

### 2.4 Preparation of solution for method validation

#### 2.4.1 Sensitivity solution

An appropriate amount of the reference solution was diluted with methanol to obtain a solution with a concentration of 5 ng/mL.

#### 2.4.2 Quantification limit solution

An appropriate amount of the reference solution was diluted with methanol to obtain a solution with a concentration of 10 ng/mL.

#### 2.4.3 Recovery solution

An appropriate amount of the nitrite standard solution was diluted with methanol to prepare a solution with a concentration of 1,000 ng/mL. The nitrite standard solution was added to rifapentine capsules (prepared as above) to achieve different final spiked concentrations: low (50 ng/mL), medium (100 ng/mL), and high (150 ng/mL). Three replicates were prepared for each concentration as recovery test solutions.

### 2.5 Preparation of nitrosation degradation solution

Rifapentine API (15 mg) was added to nitrite standard solutions at concentrations of 1, 2, 3, 4, 5, 6 and 7 g/100 g. The samples were freeze-dried and placed in an oven at 60 °C for 3 days. Then, they were diluted with 2 mL of methanol to obtain a 7.5 mg/mL solution.

For the rifapentine sample solution, 15 mg of rifapentine API was placed in a 50-mL centrifuge tube, to which 5 mL of 100 μg/mL nitrite standard solution was added, and the tube was freeze-dried. After freeze-drying, the sample was placed in an oven at 60 °C for 3 days. Then, methanol (2 mL) was added to prepare a solution with a concentration of 7.5 mg/mL. For the rifapentine control solution, 5 mL of water was used instead of the nitrite standard solution.

For the ^15^N-nitrite solution, 5 mg of ^15^N-nitrite was placed in a 50-mL volumetric flask, dissolved with deionized water, and diluted to prepare a solution with a concentration of 100 μg/mL.

For the ^15^N-nitrite-rifapentine solution, 37.5 mg of rifapentine API was placed in a 50 mL centrifuge tube, to which 5 mL of 100 μg/mL ^15^N-nitrite solution was added. The tube was then shaken and freeze-dried for 24 h. After freeze-drying, the sample was placed in an oven at 60 °C for 7 days. Methanol (5 mL) was added to prepare a solution with a concentration of 7.5 mg/mL.

### 2.6 Chromatographic conditions

#### 2.6.1 Ion chromatography detection method (nitrite detection method pump 1)

An anion exchange column (Dionex IonPac™ AS11-HC) equipped with a conductivity detector was used in suppressed conductivity detection mode. The column temperature was maintained at 30 °C, and the eluent was generated by an, EG eluent generator. Gradient elution was performed as described in Table 1. The column-switching online matrix elimination method was employed for sample injection at an injection volume of 50 μL.

**TABLE 1:** Ion chromatography eluent gradient (pump 1).

Time (min)	Eluent concentration (mM)	Flow rate (mL/min)
0.0	7	1.0
15.0	7	1.0
15.0	40	1.0
28.0	40	1.0
28.0	7	1.0
32.0	7	1.0

Retention time for nitrite (NO2-) is approximately 15.0 min.

#### 2.6.2 Column-switching online matrix elimination method

**FIGURE 3:**
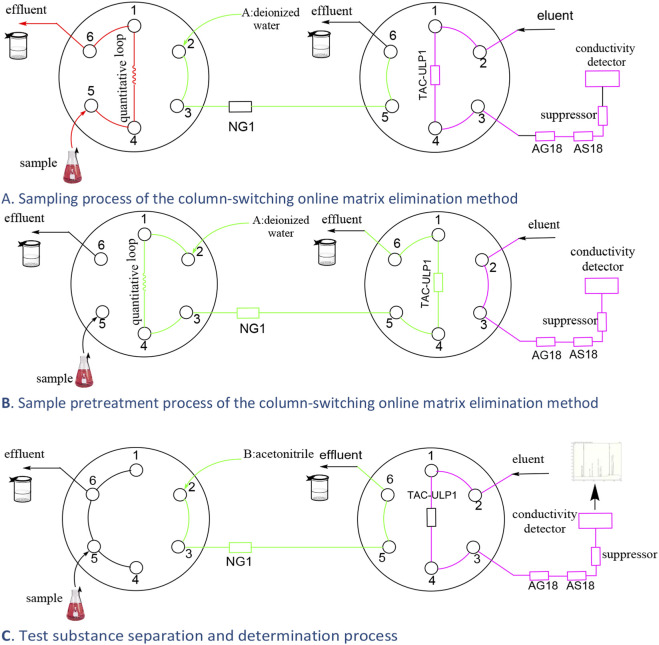
Column-switching online matrix elimination process. **(A)** Sampling process of the column-switching online matrix elimination method. **(B)** Sample pretreatment process of the column-switching online matrix elimination method. **(C)** Test substance separation and determination process.

The column-switching online matrix elimination method was used to separate the main component—rifapentine—from NO_2_^−^. The injection state, in which the sample was loaded into the sample loop, is shown in Figure 3A; the six-port valve was switched to the state shown in Figure 3B. The sample in the loop was introduced into the NG1 column with deionized water. Owing to its hydrophobicity, rifapentine was retained in the NG1 column, whereas NO_2_^−^ was instantly eluted and retained in the AG11-HC column for enrichment. After 4 min, the six-port valve was switched to the state shown in Figure 3C, and the NO_2_^−^ retained in the AG11-HC column was eluted with the gradient eluent and analyzed using the conductivity detector. Simultaneously, the gradient pump was switched to acetonitrile to elute rifapentine from the NG1 column (Table 2).

**TABLE 2:** Ion chromatography eluent gradient (pump 2).

Time (min)	Mobile phase A (%)	Mobile phase B (%)	Flow rate (mL/min)
0.0	100	0	1.0
4.0	100	0	1.0
4.1	0	100	1.0
28.0	0	100	1.0
28.1	100	0	1.0
32.0	100	0	1.0

Pump 2A: deionized water; Pump 2B: acetonitrile.

## 3 Results

### 3.1 Validation of the ion chromatography method

#### 3.1.1 Specificity test

**FIGURE 4:**
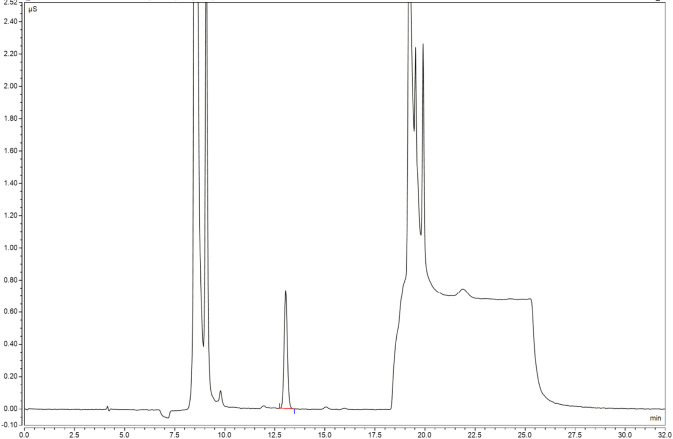
Rifapentine solvent chromatogram.

For the specificity test, 50 μL each of the blank solvent (52% methanol solution) and rifapentine capsule test solution were injected into the ion chromatograph. Chromatograms were recorded for the blank solvent, which showed no interference in its nitrite ion peak and the main component, rifapentine, which did not show any interference from impurity detection (Figure 4).

#### 3.1.2 Detection and quantification limits

**FIGURE 5:**
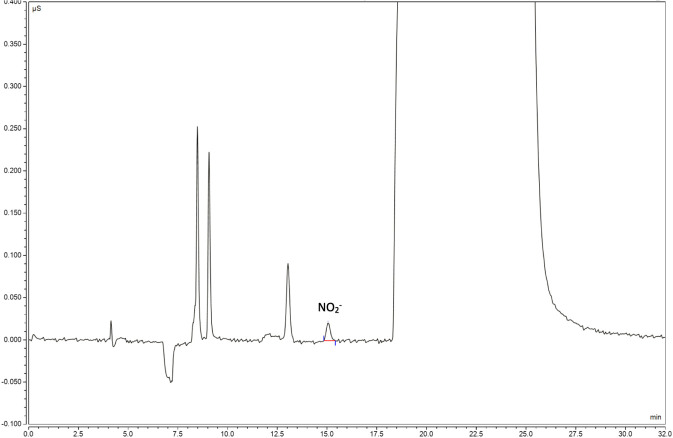
Quantification limit solutions chromatogram.

**FIGURE 6:**
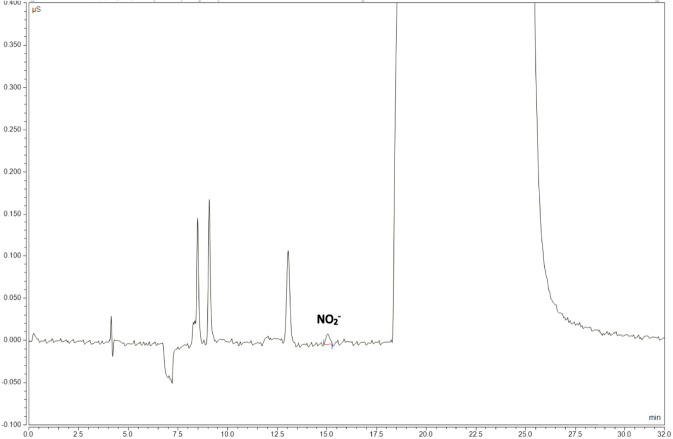
Detection limit solutions chromatogram.

The quantification (50 μL, concentration:10 ng/mL)and detection (50 μL, concentration: 5 ng/mL) limit solutions for the rifapentine capsules were injected into the ion chromatograph. The signal-to-noise ratio for the quantification and detection limit solutions were 15.0 and 3.8, respectively, which met the sensitivity requirements (Figures 5, 6).

#### 3.1.3 Repeatability

The nitrite reference solution (50 μL, concentration: 100 ng/mL) was injected into the ion chromatograph. This process was repeated five times, and the resulting relative standard deviation of the peak areas was 1.87%, indicating good repeatability.

#### 3.1.4 Linearity and range

Nitrite ion solutions (50 μL, concentration: 10–200 ng/mL) were injected into the ion chromatograph. Chromatograms were recorded, and linear regression analysis was conducted for concentration (X) versus peak area (Y). The results indicated a good linear relationship for nitrite ions for a concentration range of 10–200 ng/mL (*r* ^2^ = 0.997).

#### 3.1.5 Recovery rate

The three test solutions (concentration: 50, 100 and 150 ng/mL) showed good recovery rates of nitrite ions in rifapentine capsules. For a detailed Recovery rate Table, please refer to Supplementary Content s1.

### 3.2 Nitrosation degradation results

**FIGURE 7:**
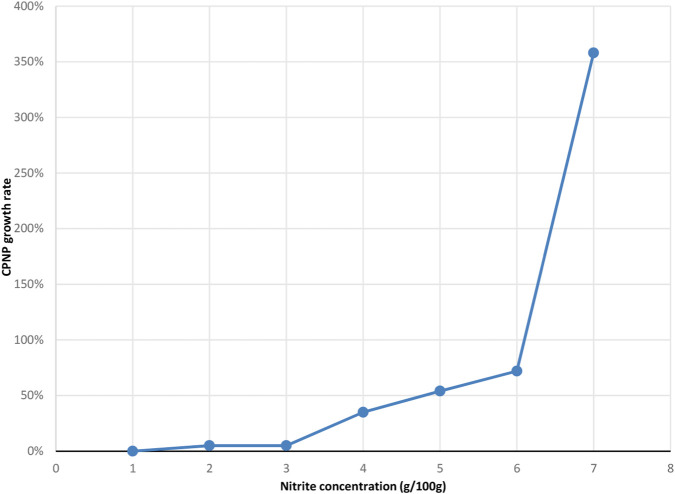
Growth curve of CPNP content under nitrosation degradation conditions.

For nitrite ion concentrations <3g/100 g, the CPNP content was not significantly different from that in the control solution. However, for nitrite ion concentrations >3g/100 g, the CPNP content increased as nitrite concentration increased (Figure 7).

### 3.3 Verification of the nitrosation pathway

**FIGURE 8:**
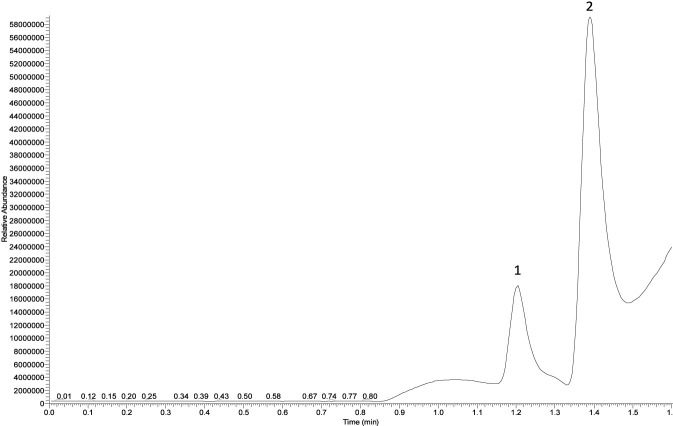
Typical chromatogram of CPNP after degradation labeling with ^15^N-sodium nitrite. Note:Peak one is ^14^N-CPNP m/z 184.1436, and peak 2 is ^15^N-CPNP m/z 185.1418.

**FIGURE 9:**
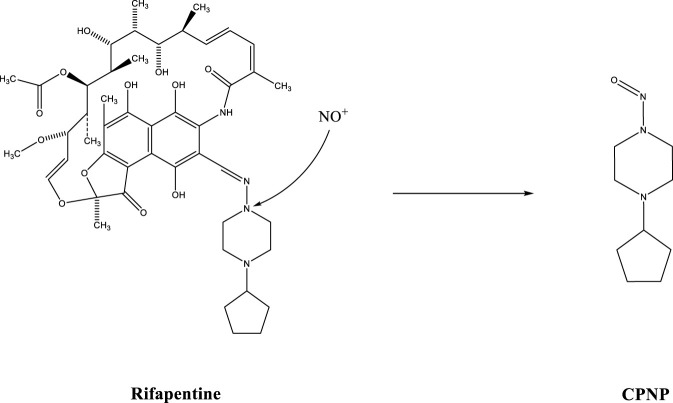
Nitrosation reaction.

The nitrosation degradation pathway was determined by adding ^15^N-nitrite to rifapentine. Compared with the unlabeled samples (1.20 min), ^15^N-CPNP was eluted at approximately 1.39 min (Figure 8). The experimental results indicated that ^15^N from sodium nitrite was present in CPNP, confirming that nitrosation reactions can generate CPNP (Figure 9). For a detailed description of the CPNP and ^15^N-CPNP detection methods, please refer to Supplementary Content s2-s3 and s4-s5, respectively.

### 3.4 Nitrite ion detection

The column-switching ion chromatography method was used to detect nitrite ions in seven batches of rifapentine capsules with varying CPNP contents (4.25–20.00 ppm). No nitrite ions were detected in the samples.

## 4 Discussion

Nitrification degradation experiments demonstrate that CPNP in rifapentine can be generated via a nitrosation pathway. This pathway was confirmed by the experiment of ^15^N isotope labeling. Therefore, the presence of nitrite ions in pharmaceuticals increases the risk of nitrosamine impurities exceeding regulatory limits, making it necessary to control nitrite ion levels in rifapentine. Conventional nitrite assays, such as spectrophotometry, electrochemistry, gas chromatography, and liquid chromatography, have various limitations. In contrast, ion chromatography excels in its efficiency, simplicity, and eco-friendliness. However, the complex matrix of rifapentine capsules and low polarity of the main component can lead to adsorption of the main component on the chromatographic column; thus, pretreatment is necessary.

In this study, we established a column-switching ion chromatography method to detect nitrite ions in rifapentine capsules. The proposed ion chromatography method avoids complex pretreatment steps and offers low detection and quantification limits, excellent linearity and reproducibility, and high recovery rates, rendering it an effective tool for controlling nitrite ions in rifapentine capsules. The validation results showed that the proposed method has good linearity, accuracy, quantification, and detection limits, and can effectively detect nitrite ions in rifapentine capsules.

## 5 Conclusion

In this study, we established a column-switching ion chromatography method to detect nitrite ions in rifapentine capsules. This method was used to verify that nitrite ions, whether present in the starting materials or remaining as residuals, can react with the main component of rifapentine capsules to form genotoxic impurities, such as 1-methyl-4-nitrosopiperazine and CPNP. This indicates that the presence of nitrite ions in pharmaceuticals increases the risk of nitrosamine impurity levels surpassing regulatory limits.

Our findings also suggest that, although nitrite ions in rifapentine formulations can increase genotoxic impurity levels, an absence of nitrite ions in rifapentine capsules under various CPNP contents does not preclude other degradation pathways contributing to CPNP formation.

This study has practical significance for the quality control of genotoxic impurities in the process of tuberculosis drug development.

## Data Availability

The original contributions presented in the study are included in the article/Supplementary Material, further inquiries can be directed to the corresponding authors.
