# Supplementary material for: Determination of nitrite in rifapentine and analysis of the formation causes of nitrosamine impurity
Source: Front Chem. 2026 Mar 17;14:1791665. doi: 10.3389/fchem.2026.1791665 (PMC13036227; doi:10.3389/fchem.2026.1791665)
Supplement: Supplementary file 1 [file Table1.doc]

**Supplementary Content**

Determination of Nitrite in Rifapentine and Analysis of the Formation Causes of Nitrosamine Impurity

*Yaodong Ping^a,d‡^,* *Yongxiang Liub,^c‡^, Nie Wenc, Bin Lib,c and Yuan Chen^*,d^ Hairuo Wen^*,c^ Ye Tian^*,c^*

1. Key laboratory of Carcinogenesis and Translational Research (Ministry of Education/Beijing), Department of Pharmacy, Peking University Cancer Hospital & Institute, Beijing 100142, China.
2. Shenyang Pharmaceutical University, Shenyang 110016, China.
3. Institute for Safety Evaluation，National Institutes for Food and Drug Control，Beijing Key Laboratory for Safety Evaluation of Drugs，Beijing 100176, China.
4. Department of Pharmacy, Peking University Cancer Hospital (Inner Mongolia Campus) & Affiliated Cancer Hospital of Inner Mongolia Medical University, Inner Mongolia Autonomous Region, Hohhot 010020, China)

**List of Contents**

| No. | Content | Page |
| --- | --- | --- |
| 1 | Table 1. Rifapentine capsule-spiked recovery rates | S1 |
| 2 | Table 2. Mass spectrometry parameters | S2 |
| 3 | Table 3. CPNP monitored ion pairs | S2 |
| 4 | Table 4. LC-MS/MS Gradient elution | S3 |
| 5 | Table 5. Liquid chromatography gradient | S4 |
| 6 | Table 6. Mass spectrometry parameters | S5 |
| 7 | Table 7. CPNP mass spectrometry monitoring information | S5 |

Table 1. Rifapentine capsule-spiked recovery rates (n = 9)

|  | Low-concentration  (50 ng/mL)  recovery (%) | Medium-concentration  (100 ng/mL)  recovery (%) | High-concentration (150 ng/mL)  recovery (%) |
| --- | --- | --- | --- |
| Replicate 1 | 93.04% | 101.34% | 98.31% |
| Replicate 2 | 87.08% | 94.72% | 99.24% |
| Replicate 3 | 92.21% | 99.02% | 96.48% |
| Mean (%) | 90.78% | 98.36% | 98.01% |
| RSD (%) | 3.55% | 3.41% | 1.44% |
| Average recovery rate = 95.72% | | | |
| Average RSD = 4.63% | | | |

1.LC-MS/MS method (CPNP detection method)

1.1.Mass spectrometry conditions

The mass spectrometry method used a triple-quadrupole linear ion trap with an electrospray ionization (ESI) source in positive ion mode and multiple reaction monitoring (MRM). Specific parameters and monitored ion pairs are listed in Tables 1 and 2, respectively.

Table 2. Mass spectrometry parameters

| Parameter | Value |
| --- | --- |
| Curtain gas | 25.0 Psi |
| Collision gas | 8 Psi |
| IS | 4000.0 V |
| TEM | 450.0 ℃ |
| Ion source gas 1 | 65.0 Psi |
| Ion source gas 1 | 45.0 Psi |
| Polarity | Positive |
| Mass spectrometry time | 0.1–4 min to MS |
|  | 4–17 min to waste |

Table 3. CPNP monitored ion pairs

| Compound | Precursor ion (m/z) | Product ion (m/z) | Declustering potential (V) | Collision energy (V) |
| --- | --- | --- | --- | --- |
| 1-Cyclopentyl-4-nitrosopiperazine | 184.000 | 154.000* | 30 | 12 |
|  |  | 98.000 | 30 | 18 |

1.2.Liquid chromatography conditions

An octadecylsilane-bonded silica column (e.g., ACE UltraCore Super C18 4.6 × 50 mm, 2.5 μm) was used. Mobile phase A consisted of 0.001% formic acid–1 mmol/L ammonium formate in water, and mobile phase B consisted of 0.001% formic acid–1 mmol/L ammonium formate in methanol. Gradient elution was performed as shown in Table 3, with a column temperature of 35°C and injection volume of 3 μL.

Table 4. LC-MS/MS Gradient elution

| Time (min) | Mobile phase A (%) | Mobile phase B (%) | Flow rate (mL/min) |
| --- | --- | --- | --- |
| 0.10 | 60 | 40 | 0.5 |
| 2.00 | 60 | 40 | 0.5 |
| 6.00 | 0 | 100 | 0.5 |
| 6.01 | 0 | 100 | 0.8 |
| 12.00 | 0 | 100 | 0.8 |
| 12.10 | 60 | 40 | 0.5 |
| 17.00 | 60 | 40 | 0.5 |

2.LC-HRMS method (^15^N-CPNP detection method)

2.1.Liquid chromatography conditions

An octadecylsilane-bonded silica column (e.g., ACE UltraCore Super C18 4.6 × 50 mm, 2.5 μm) was used. Mobile phase A consisted of 0.001% formic acid–1 mmol/L ammonium formate in water, and mobile phase B consisted of 0.001% formic acid–1 mmol/L ammonium formate in methanol. Gradient elution was performed as shown in Table 5, with a column temperature of 35°C and injection volume of 10 μL.

Table 5. Liquid chromatography gradient

| Time (min) | Mobile phase A (%) | Mobile phase B (%) | Flow rate (mL/min) |
| --- | --- | --- | --- |
| 0.10 | 60 | 40 | 0.5 |
| 2.00 | 60 | 40 | 0.5 |
| 6.00 | 0 | 100 | 0.5 |
| 6.01 | 0 | 100 | 0.8 |
| 12.00 | 0 | 100 | 0.8 |
| 12.10 | 60 | 40 | 0.5 |
| 17.00 | 60 | 40 | 0.5 |

2.2.Mass spectrometry conditions

The mass spectrometry method followed the FDA-published LC-HRMS method using an electrospray ionization (ESI) source in positive ion mode and parallel reaction monitoring (PRM). Specific parameters are listed in Table 5, and the mass spectrometry monitoring information is listed in Table 6.

able 6. Mass spectrometry parameters

| Parameter | Value |
| --- | --- |
| Sheath gas flow rate | 55 |
| Aux gas flow rate | 15 |
| Sweep gas flow rate | 0 |
| Spray voltage | 3.5 kV |
| Capillary temperature | 300℃ |
| S-lens RF level | 0 |
| Aux gas heater temperature | 300℃ |
| Polarity | Positive |
| Mass spectrometry time | 0–3.0 min |
| Normalized collision energy (NCE) | 30 |
| Isolation window | 1 m/z |
| Microscans | 1 |
| Resolution | 70000 |
| AGC target | 1e6 |
| Maximum injection time | 100 ms |

Table 7. CPNP mass spectrometry monitoring information

| Compound | Precursor ion (m/z) | Retention time (min) | Mass deviation (ppm) | Product ion (m/z) |
| --- | --- | --- | --- | --- |
| CPNP | 184.1436 | 1.35 | −4.46 | 154.1471, 98.0973, 86.0848 |
| ^15^N-CPNP | 185.1418 | 1.35 | 1.76 | 154.1470, 98.0973, 86.0848 |
